# Injuries among Amish children: opportunities for prevention

**DOI:** 10.1186/s40621-019-0223-x

**Published:** 2019-12-17

**Authors:** Stephen Strotmeyer, Abigail Koff, Joshua N. Honeyman, Barbara A. Gaines

**Affiliations:** 1 UPMC Children’s Hospital of Pittsburgh, 4401 Penn Avenue, Faculty Pavilion, 07-63, Pittsburgh, PA 15224 USA; 2 Department of Pediatrics, University of Washington Affiliated Hospitals, Seattle, WA 98195 USA; 3 Hasbro Children’s Hospital, Warren Alpert Medical School of Brown University, 2 Dudley Street, Suite 190, Providence, RI 02905 USA

**Keywords:** Amish, Injury, Trauma, Pediatric

## Abstract

**Objectives:**

The purpose of this study was to examine the injury risk patterns among Amish children, many of whom may be exposed to uncommon injuries and limited access to care due to their agrarian lifestyle and remote communities.

**Design:**

Retrospective Chart Review.

**Methods:**

With IRB approval, we performed a retrospective review of Amish patients age ≤ 12 years presenting to a level I pediatric trauma center between January 1, 2005, and December 31, 2015. Data abstracted from the institutional trauma registry and electronic medical record were analyzed using descriptive statistics and univariate/multivariate analysis.

**Results:**

One hundred eighty-three Amish children were admitted, and 2 died from injuries. Patients were 72.1% male; the median age was 5 (IQR 3–8); median injury severity score (ISS) was 9 (IQR 4–14), Most injuries were the result of blunt force trauma (91.8%). The most frequent mechanisms were falls (42.6%), followed by animal-related (15.3%), and buggy (12.5%). Most injuries occurred at home (44.4%) or on a farm (33.9%). Hay hole falls were a unique source of injury with a high ISS (12; IQR 6–17). The overall median length of stay (LOS) was 2 days (IQR 1–3), with animal-related injuries associated with the longest LOS (3 days; IQR 1–4.75).

**Conclusions:**

The majority of injuries among Amish children are due to falls. Hay hole falls and animal-related injuries result in the highest ISS and longest LOS. These findings identify the farm as a potential target for culturally appropriate interventions for risk modification.

## Introduction

Trauma is the leading cause of death in children older than 1 year (Center for Disease Control and Prevention 2015), and hospitalizations for pediatric trauma patients contribute over $6 billion to medical expenditures in the United States [“Children’s Safety Network Report on Preventing Adolescent Injury: The Role of Health Plans.”] (Jones 1990). Currently, there is limited data in the literature on the etiology and impact of trauma in the pediatric Amish community.

Pennsylvania and Ohio are home to the largest collection of Amish settlements in the United States, with an estimated 97,000 Amish children and adults. Pennsylvania alone is home to 3 of the 12 largest Amish Settlements, with Lancaster County home to the largest settlement in the country at an estimated 36,920 (Young Center for Anabaptist and Pietist Studies 2017). While there is a range of cultural expression within the larger Old Order Anabaptist community, Amish beliefs often include avoidance of technology, conservative dress, and lifestyle, and adherence to traditional farming practices (Rohrer and Dundes 2016). The Amish largely avoid allopathic health care and health insurance, believing the latter undermines the accountability of the community (Kraybill et al. 2013).

Previous studies have investigated mechanisms of injury in the Amish community, identifying the rural agrarian lifestyle and utilization of horse-drawn buggies as unique mechanisms of injury in this population (Vitale et al. 2006). Previous pediatric studies focused on farm-related injuries established that children in agrarian communities often assume responsibility for farming and animal husbandry tasks, placing them at risk for potential work-related injury at a young age (Tevis 1994). Also, the farm and barn environments are often sites of play and recreation, increasing risk exposure (Fisher et al. 2001). However, even within agrarian communities, Amish children exhibit a unique spectrum of injury given cultural limitations on usage of technology and farming, as well as decreased access to care and health insurance (Hubler, Hubcey, 2002).

One study focusing on the pediatric Amish community specifically, identified falls as the leading cause of injury, specifically through hay holes (Vitale et al. 2006), prompting further studies (Engbrecht et al. 2016) and interventions (Batra et al. 2018). Haylofts are storage areas on the second story in barns in which hay is kept, and hay holes are holes in the floor of the lofts utilized to drop hay to the animals below.

**Fig. 1:**
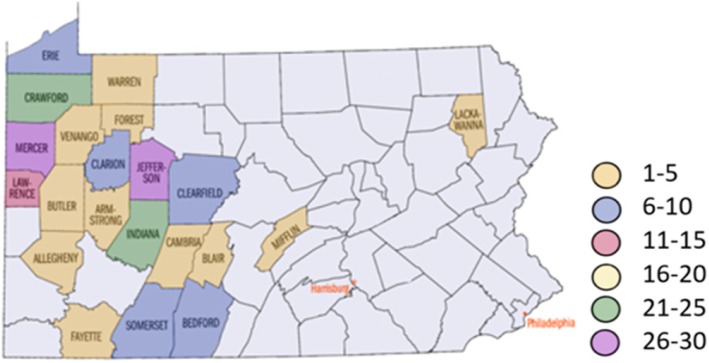
Geographic Distribution of Amish Trauma Patients

As a stand-alone children’s hospital, UPMC Children’s Hospital of Pittsburgh has a large catchment area overlapping large Amish populations (Fig. 1.), we reviewed our experience with pediatric trauma in the Amish population, focusing on the mechanism and outcome of the injury.

## Methods

Following approval from the Institutional Review Board of the University of Pittsburgh School of Medicine (PRO16100577), a retrospective review of the inpatient medical record and the institutional trauma registry was performed. We identified all Amish patients aged 12 years and younger admitted to Children’s Hospital of Pittsburgh for traumatic injuries between January 1, 2005, and December 31, 2015.

**Fig. 2:**
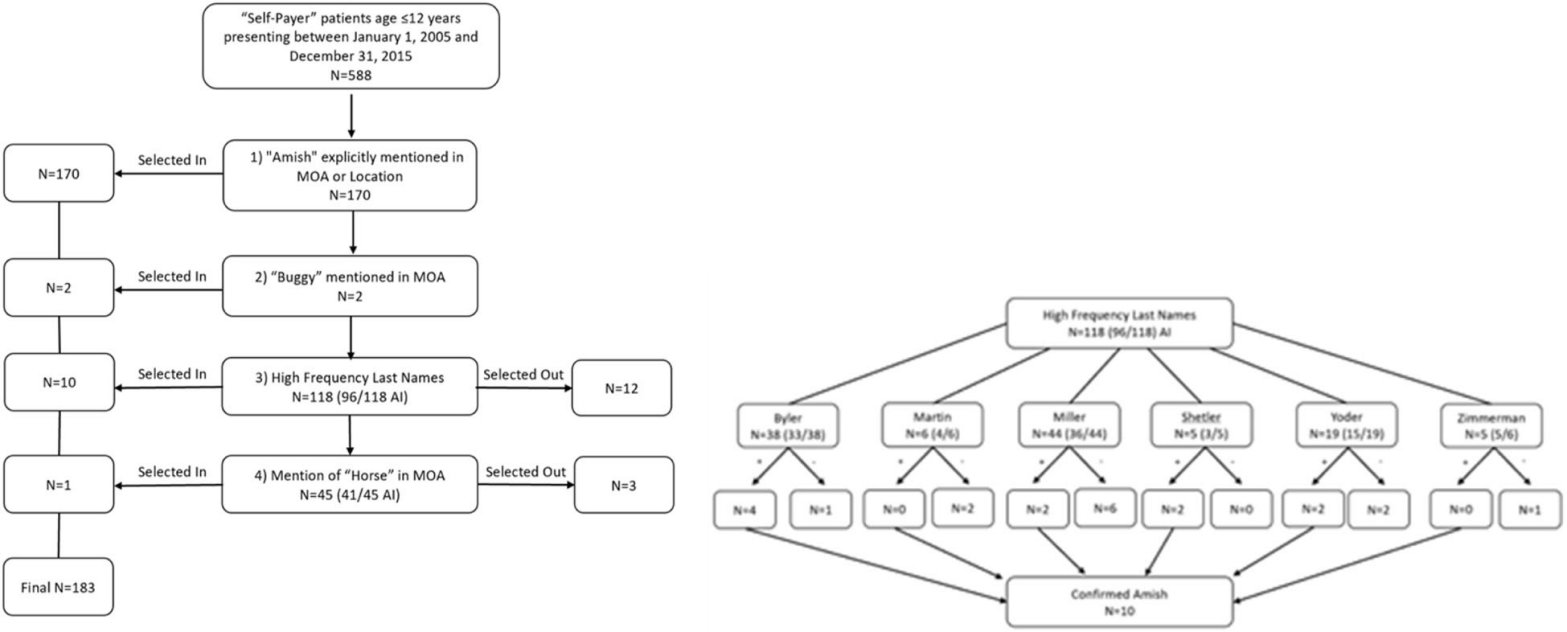
Selection Criteria: If patient met criteria for steps 3 or 4, chart review was completed for mention of “Amish”. Patients were excluded if there was no mention of “Amish” or if an alternative religion was explicitly mentioned. AI: Already included in prior screen. MOA: Mechanism of action (of injury)

From an initial cohort 588, patients were screened for inclusion into the study through a series of selection criteria (Fig. 2.) including mention of “Amish” in either mechanism of injury or location, mention of “buggy”, high frequency last names, and mentions of “horse”, “farm”, or “gun”. Patient charts were then checked for the religion section of “Patient Information”. If it stated Amish, they were ruled in. If it stated any other specific religion, they were ruled out. If it said unspecified or was blank, History & Physical Exam and progress notes were checked for a mention of Amish in social history or assessment.

Data abstracted from the trauma registry and electronic medical record included: age, sex, past medical/surgical history, date of injury, time of injury, time from injury to presentation for care, postal code where injury occurred, location where injury occurred (e.g. home, farm, road), location of first medical care, mechanism (fall, MVC, animal, buggy, pedestrian, bicycle, machinery, recreational vehicle), documented use of protective equipment (if applicable), activation trauma level, injuries, injury severity score, initial GCS, radiologic studies obtained (trauma series, number of CT scans, number of MRI), hospital admission, length of stay, ICU admission, operative procedures, total hospital charges, disposition (e.g. home, rehab), and residual disability.

Prior to analysis, all data were exported to an electronic spreadsheet for further data cleaning and coding. Descriptive statistics were used to describe demographic information. That is, frequencies and proportions were reported for categorical variables, while the means and standard deviations or 95% confidence intervals (CI) were reported for continuous variables. Paired student t-tests were used to determine if there were any significant differences between the observations. All statistical analyses were performed using SPSS Version 25 [IBM Corp. Released 2017. IBM SPSS Statistics for Windows, Version 25.0. Armonk, NY: IBM Corp.].

## Results

Between January 1, 2005, and December 31, 2015, 183 Amish children were evaluated for traumatic injury. Children ranged in age from 1 month to 12 years (Mean 5.43, Median 5, IQR 3–8). Patients were 72.1% Male and 27.9%, Female (Table 1.).

**Table 1:** Demographic Counts

Gender	
Male	132
Female	51
Age (in years)	
< 1	7
1–5	102
6–10	55
11–12	19

While the catchment area for trauma referrals includes urban and rural environments throughout western Pennsylvania, eastern Ohio, West Virginia, and western New York, over 50% of identified Amish patients came from only four counties: Mercer (14.8%), Jefferson (14.2%), Indiana (13.1%), and Crawford (13.1%).

**Fig. 3:**
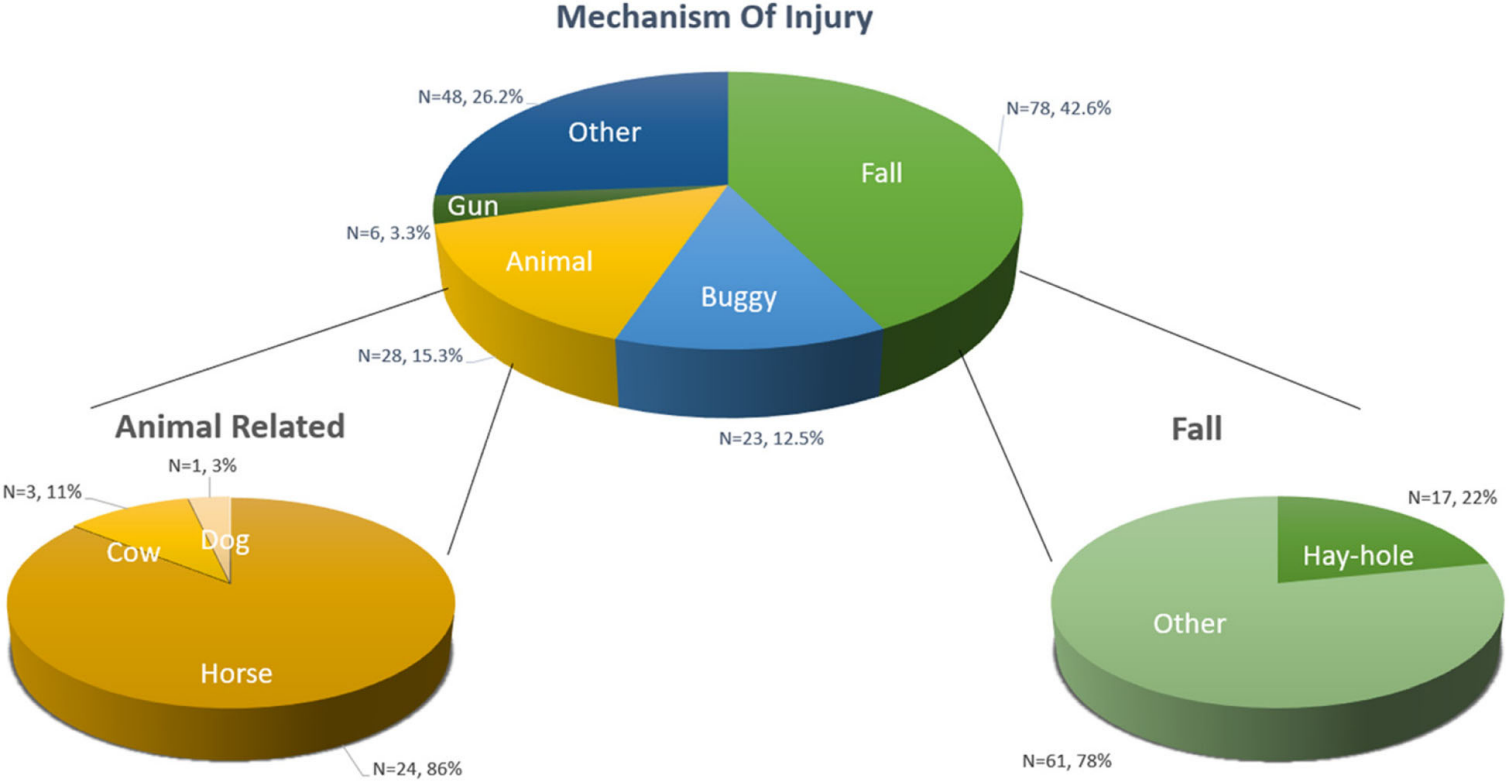
Mechanism of Injury

**Fig. 4:**
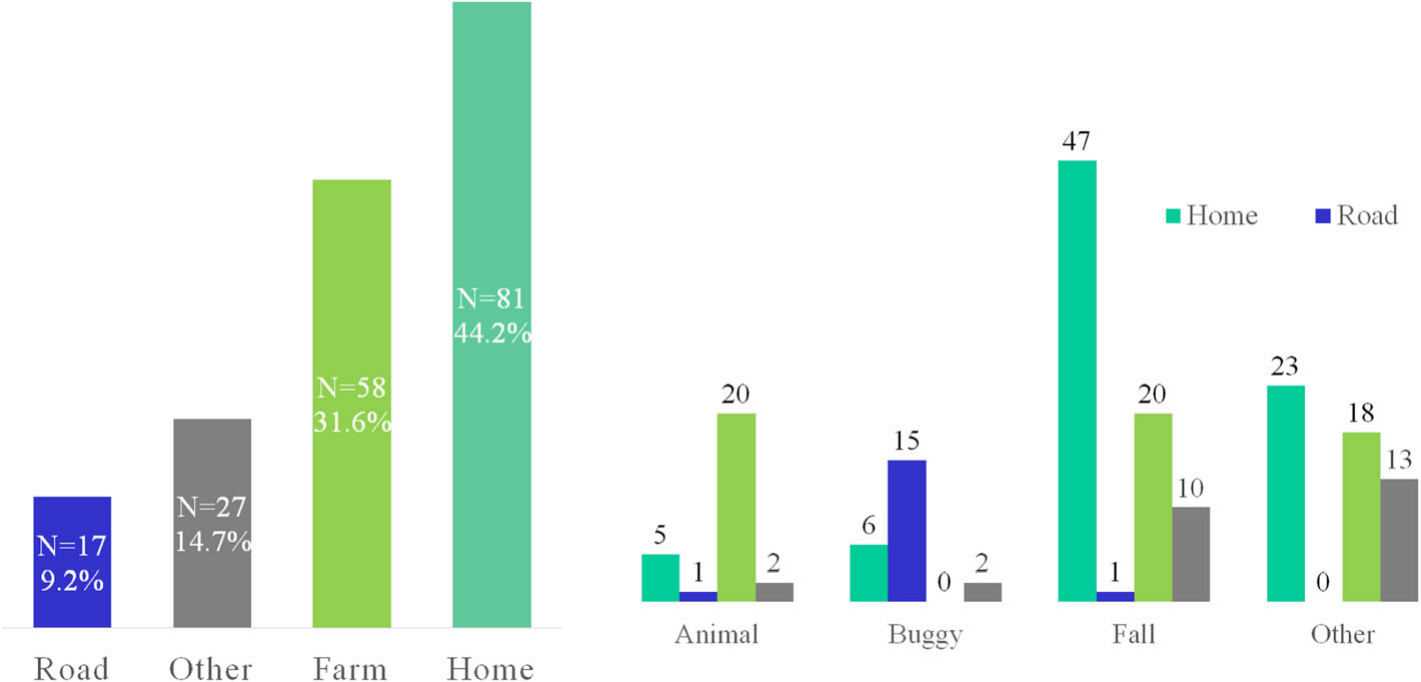
Number of Injuries by Location and Mechanism

Most injuries occurred at home (44.2%) or on a farm (31.6%) with an additional 9.2% occurring on the roadway. (Fig. 3.) The most frequent mechanisms were falls (42.6%), followed by animal-related (15.3%), buggy (12.5%), and gun-related (3.3%) (Fig. 4).

Fourteen percent of patients met the criteria for the highest level of trauma activation. Of the 26 Level I activations, 8 were animal-related in nature, 3 were the result of hay hole falls, and 6 were directly related to farm equipment. The median injury severity score (ISS) was 9.58 (IQR 4–14; range 1 to 35). While the majority of patients (85.8%) had a normal Glasgow Coma Score (GCS), the next most frequent GCS was 3 (7.1% of patients). There did not seem to be a strong correlation between GCS and ISS however, with the 3 patients with the highest ISS (34–35) having a GCS of 14 to 15. Interestingly, the 2 fatally injured patients had ISS of 34 and 26, both with a GCS of 3.

Median in-hospital length of stay was 2 days (IQR 1–3; maximum 44 days). Eighty-six patients stayed 1 day in the hospital, with 20 patients staying greater than 5 days, and only 12 patients staying greater than 10 days in the hospital. Fifty-one patients (27%) were admitted to the intensive care unit, with 20.7% (38 patients) staying from 1 to 3 days. Only 5 patients had an ICU stay longer than 10 days (maximum 33 days). Of those patients admitted to the ICU, 30 (58.8%) had a skull fracture, and 39 (76.5%) had a CNS injury. Of the ICU patients, 15 (29%) suffered injuries to the lungs, and 11(21.6%) suffered axial orthopedic injuries. Mechanism of injury (MOI) for ICU patients included falls (*n* = 21; 41.2%), of which 7 (13.7%) were associated with a hay hole. The next most frequent MOA for ICU stays was animal-related injuries (*n* = 11; 21.5%).

Most patients were discharged home (95.6%), and 6 patients (3.3%) were discharged to a rehabilitation center. Two patients died of their injuries: a GSW to the head with an initial ISS of 26, and a blunt head injury with an ISS of 34.

Hay hole falls were a unique source of injury with a high ISS (12; IQR 6–17) (Table 2.) The most commonly injured organ system was neurological with 14 (87.5%) injuries, of which 12 (85.7%) had associated skull fractures. Of these patients, 7 (43.7%) patients spent at least 1 day in the ICU. All patients had a hospital LOS less than 3 days, with a majority (13, 81.2%) staying 2 days or less.

**Table 2:** Number of Injuries by Location and Mechanism

Mechanism of Injury	ISS (IQR)	LOS (IQR)
Falls	9 (4–14)	1.00 (1.00–2.00)
Falls: Hay-hole	12 (6–17)	1.83 (1.00–2.00)
Buggy	9 (4–10)	2.00 (1.00–2.00)
Animal-Related	12 (5–16)	3.00 (1.00–4.75)

Of the 28 animal-related injuries, a majority (24) involved a horse, with 20 being the result of kicking. The majority of patients (15) were under 5 years of age. Animal-related injuries were associated with the longest LOS (3 days; IQR 1–4.75). Eleven (36.6%) patients spent at least 1 day in the ICU. There was a median ISS of 12 (IQR 5–16) with a maximum ISS of 35, the highest of all patients in the study. Of the 30-total animal-related injuries, 15 (50%) resulted in neurological injuries, 11 of which were skull fractures. Orthopedic injuries were the second most common, with 10 (33.3%) injuries.

## Discussion

The results of our study seem to be in line with the historical findings surrounding the etiology of trauma in pediatric patients. More specifically, our data support the epidemiological data surrounding the pediatric Amish population, with the most frequent mechanisms of injury being falls, followed by animal-related injuries, and buggy/transportation injuries. Most likely due to their high agrarian lifestyle, our study identified farm-related mechanisms of injury common in this population. In comparison to the non-Amish population, however, in which a majority of injuries are due to equipment (Cogbill et al. 1985), a large amount of farm-related injuries were due to falls or animals. Like a previous study of the epidemiology of pediatric Amish trauma, our study identified hay hole falls as a significant and unique source of injury with a corresponding high ISS.

Our study highlights potential areas for intervention and prevention. Both falls, and animal-related injuries make up a large portion of the injuries in the pediatric Amish population. Previous data from Penn State had identified the hay hole as a unique area for potential intervention, and with the support of the Pennsylvania Amish Safety Committee, a hay hole cover was developed that was both culturally appropriate as well as feasible for the community. Initial data from the dispersion of these covers was encouraging, with them being well received and utilized by the Anabaptist community. Our data suggest that while hay hole falls are a significant source of trauma, animal-related injuries result in a longer length of stay, identifying another potential area for intervention. Previously, multiple strategies have been aimed at pediatric farm-related injuries, but there has been little data published on the long-term effects of such intervention. One systematic review found some effectiveness in regards to short term knowledge in school-based programs and safety day camps aimed at the intervention of acute pediatric agricultural injuries, but mixed results were seen with farm-based interventions (Hartling et al. 2004). This study also found that retention and knowledge acquisition improved if accompanied by education on pediatric developmental stages and/or a farm visit from a dedicated safety specialist.

Any potential intervention strategy would necessitate a partnership with the Amish and Anabaptist communities. The Trauma and Acute Care Surgery department at Lancaster General Health has piloted interventions such as Annual Farm Safety Days and the Amish Safety Committee, in which Amish leaders meet with a member of the Benedum Trauma Program from Children’s Hospital of Pittsburgh, that could serve as potential guides to interventions for our own communities of Amish patients.

### Limitations

There are many limitations to our study. As a level 1 trauma center, our patient population is often higher acuity and thus may not only underestimate the number of pediatric traumas but also may not illustrate alternative mechanisms not seen at our institution due to their lower acuity. As a quaternary care center, our patient population is more severely injured, and thus may not be the most complete picture of the spectrum of severity of traumatic injuries. However, it is likely that our data are generalizable to the larger Amish community given not only the large percentage of Amish patients seen at our center but also the fact that our findings appear to be in line with findings of previous institutions. However, a multi-institution study or a larger systematic review after further studies have taken place would aid in remedying the small sample size, single geographical area, and possible selection bias.

The methods of selection introduce bias. Since at present there is no reliable way to select Amish patients from the trauma registry, our study utilized a series of “select in” methods to construct our data population. Our use of “Self-Payer Insurance” as the primary selection criteria is reasonable, however, given it has been established previously that the Amish community generally do not carry health insurance, and instead pay out of pocket, thus labeled in our system as “self-payer”.

## Conclusion

The majority of injuries among Amish children are due to falls. Hay hole falls and animal-related injuries result in the highest ISS and longest LOS. These findings identify the farm as a potential target for culturally appropriate interventions for risk modification.

We hope to use the data collected from this study to inform future studies, including outreach and educational interventions aimed at reducing the incidence of traumatic injuries and mitigating the severity of injuries that occur.

## Data Availability

Study data for the research resides at the Benedum Pediatric Trauma Program in the Division of Pediatric General and Thoracic Surgery at UPMC Children’s Hospital of Pittsburgh, University of Pittsburgh.

## Abbreviations

AI: Already included in previous inclusion criteria
CHP: Children’s Hospital of Pittsburgh
GCS: Glasgow Coma Scale
ICU: Intensive Care Unit
IQR: Interquartile Range
ISS: Injury Severity Scale
MOA: Mechanism of Action
MVC: Motor Vehicle Collision
